# Differential Impact of Education Level, Occupation and Marital Status on Performance of the Papanicolaou Test among Women from Various Regions in Brazil

**DOI:** 10.31557/APJCP.2019.20.4.1037

**Published:** 2019

**Authors:** Saionara Açucena Vieira Alves, Albert Schiaveto de Souza, Mathias Weller, Adriane Pires Batiston

**Affiliations:** 1 *Postgraduate Program in Public Health, State University of Paraíba (UEPB), Campina Grande- Paraíba,*; 2 *Institute of Biosciences,*; 3 *Instituto Integrado de Saúde, Federal University of Mato Grosso do Sul (UFMS), Campo Grande- Mato Grosso do Sul, Brazil. *

**Keywords:** Cervical cancer, prevention behaviour, Papanicolaou test

## Abstract

**Background:**

In Brazil, little is known regarding the underlying causes of differences among populations regarding socio-economic variables that affect women’s cervical cancer screening behavior. The present study focused on socio-economic variables that affect women’s performance of the Papanicolaou test, comparing two distinct Brazilian populations.

**Methods:**

We collected data regarding performance of the Papanicolaou test and socio-economic variables from 559 women in Mato Grosso do Sul (MS), in the Central East region, and 338 women in Paraíba (PB), in the Northeast region of Brazil. Nominal logistic regression modeling was performed to identify independent variables for both groups of data.

**Results:**

Of the women interviewed from MS and PB, 116 out of 599 (19.37%) and 94 out of 338 (27.81%), respectively, had not performed the Papanicolaou test within the last three years (p = 0.025). Low educational level characterized 570 (95.16%) and 203 (60.06%) of women from MS and PB, respectively (p = 0.000). Women in PB who had a low educational level and were unemployed had a 2.96-fold (OR = 0.338; 95% CI: 0.121 - 0.939) and 2.40-fold (OR = 0.416; 95% CI: 0.199 - 0.869) lower chance, respectively, to have performed the Papanicolaou test ≥ three times, or once within the last three years (p = 0.029; p = 0.014). The chance of women in MS who did not live in a stable relationship to have performed the test ≥ three times was 1.79-fold (OR = 0.560; 95% CI: 0.348 – 0.901) lower compared to women who reported a stable relationship (p = 0.039).

**Conclusions:**

High educational level, employment, and having a stable interpersonal relationship positively associated with performance of the Papanicolaou test among women in PB and MS. Despite having predominantly a low educational level, women in MS performed the Papanicolaou test more frequently than those in PB.

## Introduction

In 2012, cervical cancer was diagnosed in about 528,000 women, and contributed to about 266,000 deaths from cancer (Ferlay et al., 2015). It is the fourth most common cancer among women worldwide. About 85.0% of the incidence and 87.0% of all death cases occur in developing countries (Finocchario-Kessler et al., 2016; Franco et. al. 2017). In Brazil, the largest Latin American country, in the year 2017, there were 16,340 expected new cases of cervical cancer (INCA, 2016). The mean of 19.49 and 20.72 new cases per 100,000 women in the Northeast and Central East regions of Brazil, respectively, was higher than the 15.85 new cases in the country as a whole (INCA, 2016). In both regions, death rates between 2011 and 2015 were also higher than the average of 5.79 per 100,000 women for the country as a whole (INCA, 2015). In the Northeast and Central East regions of Brazil, cervical cancer led to 6.41 and 6.61 deaths per 100,000 women (INCA, 2015).

Since the 1940s, when the first results regarding cervical smear tests were published, the Papanicolaou test, or Pap smear test, has been established as the gold standard for early detection of cervical cancer (Vilos, 1998). Pap smears can help to detect pre-malignant states of cervical cancer, resulting in a higher chance of cure and lower mortality rates (Chang et al., 2017; Finocchario-Kessler et al., 2016; Labeit et al., 2013). Since performance of this ambulatory low-cost test is simple and fast, it has been recommended for cervical cancer screening programs in many countries (Fernandes et al., 2009, Soneji and Fukui, 2013; Ricardo-Rodrigues 2015). Several previous studies regarding adherence to public cervical cancer screening programs focused on socio-economic variables such as the woman’s income, education level, health insurance status, and the effect of medical recommendations (Dinshaw et al., 2007; Wang et al., 2010; Parke et al., 2011; McFarland, 2013; Soneji and Fukui, 2013; Kristensson et al., 2014; Menvielle et al., 2014; Roman et al., 2014; Mermedo- Carrasco et al., 2015; Ricardo- Rodrigues et al., 2015; Rifai and Nakamura, 2015; Chang et al., 2016; Farzaneh et al., 2017; Kelly et al., 2017; Salem et al., 2017).

Brazil does not have an organized public program of cervical cancer screening by invitation. Instead, women who visit public health service centers are invited opportunistically for screening (Derchain et al., 2016; Franco et al., 2017). The Brazilian Ministry of Health recommends the Pap smear for about 55 million women aged 25 - 64 years (INCA, 2017). Regarding the time interval of screening, two initial annual tests are recommended. If these tests are negative, a new test in every third year is recommended (INCA, 2017). 

Regarding socio- economic variables that affect women’s screening behavior, several previous Brazilian studies focused on public databases without explaining causal factors of differences between distinct populations (Novaes et al., 2006; Correa et al., 2012; Martínez-Mesa et al., 2013; Amorim and Barros, 2014; Filha et al., 2016; Barbosa, 2017). Other studies focused on single local populations of different regions in Brazil (Muller et al., 2008; Fernandes et al., 2009; Albuquerque et al., 2009; Gasperin et al., 2011; Albuquerque et al., 2014; Oliveira et al., 2014). Taken together, these studies established that education, occupation status, and relationship status were important socio-economic variables that affected women’s cervical cancer screening behavior. There are no studies that analyzed underlying causal differences between populations regarding the impact of these variables on Pap smear performance.

High incidence and mortality rates in the Northeast and Central East region highlight the need to better understand socio-economic variables that affect women’s screening behavior. As the Pap smear can help to reduce burden of disease, it is important to understand the motivations for women’s screening behavior, especially avoidance, as a basis for better guidance of public health campaigns. We addressed the question of how variables affect women’s Pap smear performance in two distinct populations. The study aimed to elucidate the possible differences regarding screening behavior of these women, and to identify the underlying socio-demographic variables in both populations.

## Materials and Methods

*Ethics approval*

Both sampling protocols of this study were reviewed and approved by the Brazilian National Research Ethics Committee (CAAE Plataforma Brasil: 63089416.0.0000.5187 and CEP/UFMS nº 1719). Informed verbal consent was obtained from each participant.

*Location of the study*

Data for the present cross-sectional study were obtained from Campo Grande, the capital of the state of Mato Grosso do Sul (MS) in the Central East region, and from Campina Grande, the second largest urban center in the state Paraíba (PB), in the Brazilian Northeast region. The air-travel distance between Campo Grande and Campina Grande is 2,496 km (1,551 miles).

Campo Grande (MS) has 786,797 inhabitants and is situated inland about 880 km away from the closest point at the Atlantic coast (IGBE, 2010). The Human Developmental Index is 0.784. The per capita income is R$1,089.37 (~$339 US). The Family Health Strategy (“Estratégia de Saúde da Família”), a priority project for the organization of primary health care in Brazil, covers potentially 34.35% of the whole population (Ministério da Saúde, 2017). Campina Grande (PB) has 385,213 inhabitants and is situated 125 km from João Pessoa, the capital of PB on the Atlantic coast. The Human Developmental Index is 0.720 and the per capita income is R$630.03 (~$196.00 US). The Family Health Strategy covers potentially 83.47% of the entire population (Ministério da Saúde, 2017). 

*Study population*

Women were eligible for the study if they did not have any type of cervical, ovarian or breast cancer. Women aged 40 and older receive an official recommendation to participate on the public mammography-screening program (INCA, 2017). Therefore, the present study focused exclusively on women aged 40 - 64 years.

In MS, data were obtained from 599 women who exclusively used public health care providers. Women were identified in registers of 29 Family Health Strategy Units, and were visited at home for data sampling by one of the authors. Data sampling was performed between January and December 2013. In PB data, were obtained from 338 women. Of these, 104/338 (26.3%) used public and private health care providers. Data were obtained by one of the authors in waiting rooms of two public health care centers. There were no differences between data of women from both health care centers. Data sampling was performed between March and October 2017.

*Questionnaires and measures*

In both MS and PB, data sampling was performed by interviews. In MS, a structured questionnaire was applied. Interviews performed in PB were based on a modified structured questionnaire developed in previous studies (Freitas and Weller, 2016a; Freitas and Weller, 2016b). Both questionnaires were subdivided into the following sections: 1) socio-economic characteristics; 2) reproductive and health characteristics, including information regarding previous biopsies, breast, ovarian and cervical cancer of the participant and first-degree relatives; 3) adherence to screening programs, including clinical breast examination, mammography, and the Pap smear test; and 4) information regarding use of health services.

Educational levels were defined as follows: elementary school with duration of nine years was defined as “Low”; middle school with duration of 12 years was defined as “Middle”; higher educational levels were defined as “High”. Income was sampled from PB, but not from MS. Income was defined as minimum wage and multiple values of the minimum. In 2017, the minimum wage was R$937.00/month. In December 2017, this value was equivalent to about $290.00 US/month. The following definitions were applied: ≤ 1 x minimum wage = low income; > 1 x minimum wage and ≤ 2 x minimum wage = intermediate income; > 2 x minimum wage = high income. Ethnic origin was based on self-reporting by interviewed women.

Women were asked about their adherence to recommendations by the public screening program. If asked about Pap smear performance, the following options were distinguished: never, sometimes, every year, and every other year.

*Statistical analysis*

Pearson’s Chi-Square (χ^2^) test was applied to compare categorized variables. The t-test was applied to compare continuous parametric variables. Results of multinomial logistic regression were presented as adjusted odd ratios (OR), 95% confidence interval (95% CI) and *P-values*. P values of regression analyses were calculated using likelihood ratio tests (PLRT) for each independent variable. Significant variables from univariate regression analysis were used for regression modeling: variables with significance level less than 0.2 in the univariate analysis were entered into the model. Then, variables with significance level less than 0.05 were kept in the model. Backward selection was used when significant variables were selected. The final model was tested for fitness using the likelihood ratio test. All statistical analyses were performed using SPSS STATISTICS™ software (SPPS; IBM company; version 24).

## Results

Comparison of data revealed differences between women from MS and PB (Table 1): the mean age of women from PB and MS was 51.08 (SD = 8.89) and 52.86 (SD = 8.89) years, respectively (p = 0.003; Table 1). Of all 599 women from MS, 230 (38.40%) were aged 40 - 49 years, whereas 167 out of 338 (49.41%) women from PB belonged to this age category (p = 0.005; Table 1). High education level, defined as schooling > 12 years, was only found among 30 (8.88%) women from PB, whereas in MS, 570 (95.16%) and 29 (4.84%) women, had low and intermediate educational levels, respectively (p = 0.000; Table 1). A positive occupation status was identified for 137 (22.87%) women from MS and 107 (35.47%) from PB (p = 0.002; Table 1). Finally, women from MS performed the Pap smear more often than did women from PB (Table 1): Information obtained from women in MS revealed that 116 (19.37%) had never performed the Pap smear, whereas in PB, 94 (27.81%) women had never performed it (p = 0.025; Table 1). No significant differences were found for marital status and ethnic origin between women from MS and PB (p = 0.234; p = 0.059; Table 1).

Age-specific differences of Pap smear performance were found in both groups of women from MS and PB (p = 0.001; p = 0.000; Table 2): Compared with women aged 60 - 69 years, those from MS and PB aged 40 - 49 years, had a 3.438-fold (95% CI: 1.952 - 6.057) and 3.543-fold (95% CI: 1.658 - 7.627) higher performance rate of the Pap smear ≥ three times within the last three years (Table 2). Similarly, among women from MS and PB aged 50 - 59 years, the performance rate of the Pap smear ≥ three times, was 2.961-fold (95% CI: 1.685 - 5.202) and 3.556-fold (95% CI: 1.658 - 7.627) higher, respectively, than rates for women aged 60 - 69 years (Table 2). In addition, the chance of women in MS, aged 40 - 49 and 50 - 59 years, to have performed the Pap smear two times within the last three years, were 2.857-fold (95% CI: 1.514 - 5.393) and 2.302-fold (95% CI: 1.215 - 4.363) higher, than rates for women aged 60 - 69 years (Table 2). 

Data of education level and occupation status were heterogeneously distributed for women from PB (p = 0.011; p = 0.014), but not for women from MS (Table 2): performance of the Pap smear test ≥ three times, was 2.83-fold (OR = 0.353; 95% CI: 0.131 - 0.949) lower among women from PB with low educational level, than for women with high education level (Table 2). Women from PB who were not employed performed it once within three years, 3.03-fold (OR = 0.330; 95% CI: 0.163 - 0.671) less often than did women who were employed (Table 2). Marital status was a significant variable only for data obtained from women in MS (p = 0.002), but not for those in PB (Table 2): Women without a stable relationship performed the Pap smear ≥ three times within the period of three years, about 2.18-fold (OR = 0.458; 95% CI: 0.290 - 0.723) less often than did women who lived in a stable relationship (Table 2). 

In an age-adjusted regression model, education level and occupation status retained their heterogeneous distribution among data obtained from women in PB (p = 0.029; p = 0.014), whereas for women from MS, marital status remained the unique significant variable (p = 0.039; Table 3). In this model, women from PB with low educational level and no occupation performed the Pap smear ≥ three times and once within three years, 2.96-fold (OR = 0.338; 95%CI:0.121 - 0.939) and 2.40-fold (OR = 0.416; 95% CI: 0.199 - 0.869) less often, compared with women with high educational level and positive occupation status, respectively (Table 3). The chance of women in MS, who did not live in a stable relationship, to perform the Pap smear ≥ three times, was 1.79-fold (OR = 0.560; 95% CI: 0.348 – 0.901) lower than for women who lived in a stable relationship (Table 3). 

## Discussion

Present results indicated that high educational level, employment, and living in a stable relationship were independent variables that increased the chance of Pap smear performance among women in PB and MS. Compared with women from MS, women in PB were younger, were more often employed, lived more often in a stable relationship, and had performed the Pap smear less often during the last three years. The most different variable among both groups of women was education: more than 95% of women in MS were reported low educational level, and there was nobody in this group with high educational level, whereas in PB, about 60.0% and 40.0% of women had a low, intermediate or high educational level, respectively. This suggested that data regarding education were more homogeneously distributed among women from MS than among those from PB.

**Table 1:** Socio-Economic Variables, Performance of the Papanicolaou Test and Their Distribution among Women from the States of Mato Grosso do Sul (MS) and Paraíba (PB)

	Mato Grosso do Sul (N= 599)		Paraíba (N= 338)		P
Mean	52.86 (s = 8.45)		51.08 (s = 8.89)		0.003
Age	N	%	N	%	
40 - 49 years	230	38.40%	167	49.41%	0.005
50 - 59 years	217	36.23%	102	30.18%	
≥ 60 years	152	25.37%	69	20.41%	
Education level					
Low	570	95.16%%	203	60.06%	0
Intermediate	29	4.84%%	105	31.06%	
High	0		30	8.88%	
Occupation status					
Employed	137	22.87%	107	35.47%	0.002
Not employed	462	77.13%	231	64.53%	
Stable relationship					
Yes	376	62.77%	221	65.39%	0.234
No	223	37.23%	117	34.61%	
Ethnic origin					
European	236	39.40%	115	34.02%	0.059
Mixed ethnic background	363	60.60%	223	65.98%	
Performance of Papanicolaou test within the last three years					
Never	116	19.37%	94	27.81%	0.025
One time	110	18.36%	58	17.16%	
Two times	130	21.70%	60	17.75%	
≥ three times	243	40.57%	126	37.28%	

**Table 2:** The Impact of Single Socio-Economic Variables on Papanicolaou Test Performance within the Last Three Years, for Women from the Paraíba State (PB; N = 338) and Mato Grosso do Sul State (MS; N = 599), Respectively. Data are represented as odds ratios (OR) and confidence intervals (95%CI). Non-performance of Papanicolaou test served as reference

		One time		Two times		≥ three times		
		OR	95%CI	OR	95%CI	OR	95%CI	P
Age (years)								
40 - 49	MS	1.727	0.905 - 3.296	2.857*	1.514 - 5.393	3.438*	1.952 - 6.057	0.001
50 - 59		1.714	0.907 - 3.242	2.302*	1.215 - 4.363	2.961*	1.685 - 5.202	
40 - 49	PB	5.790*	2.161 - 15.513	1.95	0.896 - 4.248	3.543*	1.709 - 7.346	0
50 - 59		2.765	0.935 – 8.138	1.027	0.417 – 2.532	3.556*	1.658 - 7.627	
≥ 60	Ref.							
Education level								
Low	MS	1.945	0.474 – 7.978	1.127	0.353 - 3.597	0.892	0.334 - 2.384	0.632
Intermediate		Ref.						
Low	PB	3.088	0.358 - 26.669	0.471	0.141 – 1.573	0.353*	0.131 - 0.949	0.011
Intermediate		6.6	0.730 - 59.679	1.1	0.305 – 3.970	0.724	0.247 – 2.116	
High	Ref.							
Occupation status								
Not employed	MS	1.986*	1.052 - 3.750	1.444	0.813 - 2.566	1.417	0.860 - 2.337	0.196
	PB	0.330*	0.163 - 0.671	0.497	0.243 - 1.016	0.692	0.373 - 1.284	0.014
Employed	Ref.							
Stable relationship								
No	MS	0.891	0.528 - 1.504	0.649	0.390 – 1.081	0.458*	0.290 - 0.723	0.002
	PB	0.587	0.289 - 1.193	0.83	0.423 - 1.626	0.798	0.459 - 1.389	0.522
Yes	Ref.							
Ethnic origin								
European ethnic origin	MS	1.173	0.680 - 2.025	1.692	1.009 - 2.838	1.289	0.811 - 2.049	0.23
	PB	1.421	0.693 – 2.914	1.689	0.838 – 3.402	1.919	1.069 - 3.445	0.161
Mixed origin	Ref.							
Income								
Low	PB	0.537	0.073 - 3.976	0.291	0.051 - 1.663	0.383	0.075 - 1.962	0.626
Middle		0.8	0.103 - 6.191	0.34	0.056 - 2.069	0.573	0.107 - 3.059	
High	Ref.							

**Table 3:** Odds Ratios (OR) and Confidence Intervals (95%CI) in Age-Adjusted Models (p = 0.000) of Logistic Regression Represented for Women from Paraíba State (PB; N = 338) and Mato Grosso do Sul State (MS; N = 599), who Performed Papanicolaou Test within Three Years. Non-performance of Papanicolaou test served as reference

		One time		Two times		≥ three times		
		OR	95%CI	OR	95%CI	OR	95%CI	P
Stable relationship								
No	MS	0.996	0.579 - 1.711	0.786	0.463 – 1.334	0.560*	0.348 – 0.901	0.039
Yes	Ref.							
Education level								
Low	PB	3.554	0.399 - 31.694	0.508	0.150 - 1.727	0.338*	0.121 - 0.939	0.029
Middle		5.311	0.568 - 49.698	1.016	0.274 - 3.770	0.572	0.188 - 1.742	
High	Ref.							
Occupation status								
Not employed	PB	0.416*	0.199 - 0.869	0.583	0.277 - 1.228	0.952	0.497 - 1.823	0.014
Employed	Ref.							

*p ≤ 0.050

In the literature, education is one of the most-often identified independent variables: Many previous studies suggested a positive association of high educational level with performance of the Pap smear (Wang et al., 2010; Park et al., 2011; McFarland, 2013; Soneji and Fukui, 2013; Kristensson et al., 2014; Menvielle et al., 2014; Mermedo- Carrasco et al., 2015; Ricardo- Rodrigues et al., 2015; Chang et al., 2016; Farzaneh et al., 2017; Kelly et al., 2017). However, two recent studies from Jordan and Saudi Arabia, as well as a study from India, revealed no association between education and Pap smear performance (Dinshaw et al., 2007; Rifai and Nakamura, 2015; Salem et al., 2017). An increased chance of Pap smear performance for women with higher educational levels, was also reported by most authors of Brazilian studies (Novaes et al., 2006; Muller et al., 2008; Albuquerque et al., 2009; Fernandes et al., 2009; Gasperin et al., 2011; Correa et al., 2012; Martínez- Mesa et al., 2013; Oliveira et al., 2014; Filha et al., 2016; Barbosa, 2017). However, a study including 6,750 households in the state of São Paulo, and a study of 493 women from Piauí revealed that education was not significantly associated with performance of the Pap smear (Albuquerque et al., 2014; Amorim and Barros, 2014). In the present study, women from PB with low educational level tended to less often perform the Pap smear two or more times during the last three years, compared with women with high educational level. Paradoxically, in MS, many more women had low educational level, but performed the Pap smear more often than did women in PB. In MS, variables other than education determined the frequency of Pap smear performance. Despite its significant heterogeneous distribution among women from PB, the education variable did not determine absolute frequency of Pap smear test performance of Brazilian women in this study.

Women from PB who were not employed tended to perform the Pap smear test less frequently than did employed women. This finding agrees with findings from previous studies performed in Botswana, Colombia, India and France, that reported an increased chance of Pap smear performance by employed women (Dinshaw et al., 2007; McFarland, 2013; Menvielle et al., 2014; Mermedo-Carrasco et al., 2015; Kelly et al., 2017). A Chinese study, by contrast, did not show a significant association between occupation status and lifetime Pap smear performance (Wang et al., 2010). Furthermore, in the study from Rifai and Nakamura (2015), performed in Jordan, unemployed women performed lifetime Pap smear more often than did employed women. In Brazil, the few studies that included occupation status returned different results: Similar to present results, studies based on data from distinct Brazilian regions and from the state Rio Grande do Norte, Northeast Brazil, revealed that employed women had a higher chance of Pap smear performance (Novaes et al., 2006; Fernandes et al., 2009). Other previous studies, by contrast, performed in the states of Santa Catarina, South region, and Pernambuco, Northeast region, did not show an effect of occupation status on frequency of Pap smear performance (Albuquerque et al., 2009; Gasperin et al., 2011).

Present data suggested that women from MS who lived in a stable relationship performed the Pap smear more frequently than did women who did not live in a stable relationship. In the case of data from PB, the OR’s had the same tendency in univariate analysis, but differences remained insignificant. In agreement with results for data from MS, most studies that included civil status as a socio-economic variable, revealed a positive association between living in a stable relationship and Pap smear performance (Dinshaw et al., 2007; Wang et al., 2010; Park et al., 2011; McFarland, 2013; Kristensson et al., 2014; Menvielle et al., 2014; Ricardo-Rodrigues et al., 2015) A study from the US also identified living in a stable relationship as an independent variable that increased the chance of Arab women to perform Pap smear (Roman et al., 2014). Recent studies from Saudi Arabia and Iran, by contrast, did not show an association between civil status and Pap smear performance (Farzaneh et al., 2017; Salem et al., 2017). Most Brazilian studies that included civil status in their analysis also showed that women who lived in stable relationships had a higher chance to perform Pap smear than did women who did not live in a stable relationship (Novaes et al., 2006; Gasperin et al., 2011; Albuquerque et al., 2009; Albuquerque et al., 2014; Amorim and Barros, 2014; Oliveira et al., 2014; Filha et al., 2016; Barbosa, 2017). Two previous Brazilian studies, by contrast, did not show an association between civil status and Pap smear performance (Muller et al., 2008; Fernandes et al., 2009).

Interestingly, in both unique Brazilian studies that did not reveal any association of education and Pap smear performance, living in a stable relationship significantly increased the chance of its performance (Albuquerque et al., 2014; Amorim and Barros, 2014). This is comparable to women from MS in the present study: neither education nor occupation status significantly affected the chance of Pap smear performance, however, living in a stable relationship remained significant. Sexual activity and pregnancies of women who are married or live in any other kind of stable relationship may be associated with more visits to gynecologists from an early age and beyond. This, in turn, may lead more frequently to recommendation of Pap smear by physicians. However, the frequency of women who lived in a stable union was nearly the same in MS and PB. Furthermore, data from MS regarding occupation status and education were more homogenously distributed, compared to those from women from PB. Therefore, the effect of civil status may have become more important for women from MS. For women from PB, by contrast, heterogeneously distributed characteristics of education level and occupation status became more prominent than civil status.

This does not explain why women from MS performed the Pap smear than more often than women from PB. In the latter group, the frequency of women who had never performed the Pap smear was substantially higher. Differences of Pap smear performance two times within three years for women aged between 40 - 49 and 50 - 59, compared with women aged 60 were more prominent among women from MS than for those from PB. In their analysis of data from eight Latin American countries, Soneji and Fukui (2013) showed that women who visited a doctor during last 12 month before the study performed the Pap smear more frequently. The same variable was also positively associated with performance of Pap smear tests in previous Brazilian studies (Novaes et al., 2006; Albuquerque et al., 2009; Martinez-Mesa et al., 2013). Similar, in studies from Denmark and the US, contact with health service or general practitioners had an important positive effect on women’s frequency of Pap smear performance (Roman et al., 2014; Kristensson et al., 2014). One hypothesis is that women in MS visited public health services more often and the Pap smear was more frequently recommended by physicians for this reason. An alternative hypothesis is that, compared with PB, more physicians in public health services in MS had the formation to perform the Pap smear, and that it was therefore more often recommended. The fact that a high percentage of women from MS and PB performed the Pap smear within the last three years, more often than recommended (≥ three times), could mean that their prevention behaviour was influenced by recommendations of medical stuff of local health care service centres of the study. It would be interesting in this context to understand in more detail the impact of doctor visits and recommendations on prevention behavior of women who have distinct educational levels, occupation status, and civil statuses.

An important limitation of the present study was the missing data regarding doctor visits and gynecological recommendations that can trigger women’s prevention behavior. The study did not include data regarding women’s number of children and sexual activity. These are all variables that can affect prevention behavior of cervical cancer. Furthermore, data regarding income was missing for women in MS. Data were sampled during different time periods. As Brazil is a country that suffered severe economic and demographic changes during recent years, this may have led to sample bias. Applied questionnaires and sampling methods were not identical for women in MS and PB. This may have caused an additional bias of sampled data.

In summary, the present data confirmed results of previous studies, establishing education, occupation status and civil status as important variables for women’s performance of the Pap smear. In PB, higher educational level and positive occupation status were independent variables. Among women from MS, educational characteristics were highly homogenously distributed, and civil status was an independent variable. The data suggested that higher educational levels were positively associated with performance of the Pap smear in PB, but as women from MS had on average lower educational levels and performed it more frequently compared to women from PB, the impact of the variable education was not decisive for the absolute frequency of test performance. Future studies should clarify the ways recommendations from medical staff of public health care centers affect the decision of women who have different socio-economic background to perform the Pap smear.

## Conflict of interest

There is no conflict of interest.
